# Indian Society of Periodontology: Rise and reach in 17 years

**DOI:** 10.4103/0972-124X.60221

**Published:** 2009

**Authors:** Dilip G. Pol

**Affiliations:** *President 2009-2010, Indian Society of Periodontology Professor, Department of Periodontology, Government Dental College and Hospital, Mumbai - 400001, India. E-mail: ispdgpdd@rediffmail.com*


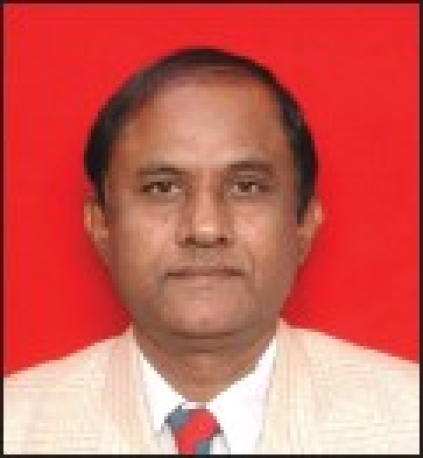


Dear Friends,

During my tenure of 14 years as the secretary of the ISP from 1994-2006 and an additional year 2007, then President Elect 2008, and President 2009, I have seen ISP rise and reach the zenith of success and popularity on not only the national but also the international front as well [Table 1].

**Table 1:** Highlights

Year	President	Place of conference	P.G. workshop	Highlights
1994	Dr. T.K. Pal	Hyderabad		ISP participation in DCI workshop for updating syllabus at Mysore
1995	Dr. Lalit Guglani	Mumbai		Society registered GS-BBD-385/95
1996	Surg. Com. J.K. Gupta	Mangalore		Introduced 2^nd^ oration in the memory of Dr. G.B. Shankalwarkar
1997	Dr.(Mrs.) R.H. Jaffer	Nagpur		Amendment to the constitution draft prepared
1998	Dr Thomas Thelly	Chennai		Marathon EC meeting for finalization of draft of amendment to the constitution
1999	Dr. (Mrs.) P.K. Bambhani	Calcutta	1) Workshop held for conducting Epidemiological survey at Lonavala	1) Amendments to the constitution passed
				2) ISP bulletin renamed as the Journal of Indian Society of Periodontology (JISP)
			2) Syllabus updated and Implantology is included in UG&PG curriculum and recommended to DCI	
2000	Dr. A. Jayakumar	Mumbai, Silver Jubilee Conf. of ISP collaboration with IAP	1^st^ Workshop at Trivandrum	Oral Hygiene day launched on 1^st^ Aug (Birthday of Dr. G.B. Shankalwarkar)
2001	Dr. B.P. Katthak	Trivandrum		ISP directory released
2002	Dr. P. K. Agrawal	Mount Abu	2^nd^ PG Workshop at Chennai	1) Exclusive ISP MERIT SHIELD is started in the name of Indian Society of Periodontology
				2) ISP participation in DCI workshop for updating syllabus at Nagpur
				3)Entire ISP recommended syllabus accepted by DCI
2003	Dr. B.V. Somayaji	Guwahati	3^rd^ PG Workshop at Belgaum	1) ISP directory published.
				2) 1^st^ Amendment to the constitution.
2004	Dr. R.N. Deshpande	Mangalore	4^th^ PG Workshop at Nashik	2^nd^ Amendment to the constitution.
2005	Dr. R. M. Kohad	Chennai,	International Event	3^rd^ Amendment to the constitution
		International Conf of ISP collaboration with IAP	1^st^ World Symposium for PG's, Mumbai, Chennai	4^th^ Category of life members added to essay competition
2006	Dr. K. Nandakumar	Pune	1) 5^th^ PG workshop at Coorg at	1) All free papers shifted to PG workshop,
			2) PG teacher Convention at Bangalore	2) Increase in prize money for essay competition
			3) ISP participation in joint Workshop of RJUHS and DCI 5-year course, Perio kept in 5^th^ year	3) DCI accepted ISP UG & PG syllabus, title changed as Periodontology and Implantology
				4) Pune AGM was adjourned without handing over
2007-April 2008	Dr. Subhash Garg	Davangere	6^th^ PG Workshop Trivendrum	1) DCI in 5-yr BDS course shifted perio to 4^th^ yr and implantology is removed from the title of subject. There was no participation of ISP during these changes
				2) JISP was not published
				3) AGM at Dharwad nominated Dr. M.M. Dayakar's name for the post of Secretary for smooth running of societ.
				4) EOGM at Mumbai handing over of office to Dr. Dayakar and all other office bearers.
2008	Dr. Pravin Kudva, Acting President	Chandigarh	7^th^ PG Workshop, Udaipur	
2009	Dr. Dilip G Pol	Dharwad	8^th^ PG Workshop, ISP:IAP Agra	1) ISP abroad – 14^th^ to 17^th^ Aug, 2009 Bankok, Thailand
				2) JISP regular issue
				3) ISP directory to be published

## CHALLENGES AHEAD

1. Perio subject exam to be kept on 5^th^ year of new BDS course.
2. JISP has to be made quarterly issue.

I have gone through a tortuous road and its ups and downs as a secretary but without all the support and backing from several people, I would not have been able to take ISP to this pedestal.

Today I am immensely happy to thank and acknowledge all the people without whom I would not achieved this success.

I would like to remember and express my gratitude towards the founding members of ISP, late. Dr. G. B. Shankwalkar and Dr.Lalit Guglani, for their dynamism and vision. They always serve as a source of inspiration for me. I thank all my office bearers during my tenure as Secretary: Dr. SA Kale, Dr. CP Boghani, Dr. PJ Chitnis, Dr. Ajay Kakar, Dr. A Kumarswamy and during my Presidential tenure: Dr. MM Dayakar, Dr. CD Dwarkanath, Dr. Pravin Kudva, Dr. Santosh Sridhar, Dr. Mathai Thomas, Dr. Anirban Chatterjee and Dr. Arunachalan.

I should not forget to acknowledge all my postgraduates and staff who were always there to help me and shore up in all situations. Without their help, it could not have been possible to run the ISP office effectively. I am profoundly thankful to Dr. Nirmala, Dr. Natasha, Dr. Ashwini, Dr. Rajesh Gaikwad, Dr. Pranita, Dr. Neha Mehta, Dr. Prajakta Nayak, Dr. Rajeev Chitguppi, Dr. Gauri Satarkar, Dr. Om Bhagele, Dr. Srinivas, Dr. Farah, Dr. Sameer Dalvi, Dr. Roshni Thakur, Dr. Akash Akinwar, Dr. Ranjit Bapat, Dr. Javed Sayyed, Dr. Vivek Dave, Dr. Yadav Rathod. Dr. Rupasi Kudle, Dr. Jyoti Jonnala, Dr. Mukesh Chute, Dr. Tanay Gunjikar and my assistant Girish Jethwa who have helped me persistently for ISP.

## PIONEERS OF 1^ST^ ISP EVENT ABROAD, BANGKOK

**Figure:**
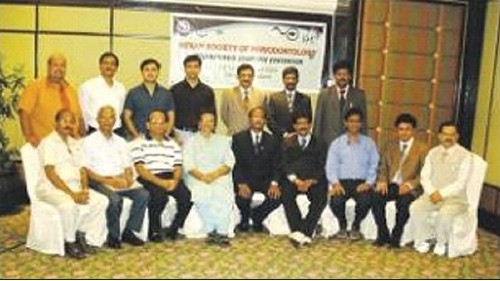
**Sitting from L to R:** Dr. Anil, Dr. TK Pal, Dr. PK Agrawal, Dr. IR D'Silva, Dr. Dilip G Pol - President, ISP, Dr. MM Dayakar - Secretary, ISP, Dr. Ajay Kakar, Dr. Rajiv Chitguppi, Dr. Pradeep Tondan. **Standing from L to R:** Dr. Saurabh Gupta, Dr. Rajendra Bane, Dr. Shushrut Prabhudesai, Dr. Kunal Sethi, Dr. PL Ravi Shankar, Dr. G Harkishan, Dr. L Chandra Sekhar

I appreciate the assistance rendered by all of them. I am also thankful to Colgate & Palmolive for sponsoring most of the ISP activities, Mr. Arekar, legal advisor and auditor A. W. Ketkar.

Last but not the least I would like to express my gratitude towards my wife Dr. Nutan Pol for being so patient to support and encourage me in this long journey. My life is blissful and contented due to my sweet and adorable daughters Dr. Samruddhi, Sukhsiddhi and Sanmati. I am thankful to them for making me comfortable and peaceful at home after my busy life after taking charge of ISP for 17 years.

This is just the beginning and the future belongs to those who believe in the beauty of dreams and conclude with my favorite hopeful sher -

Ek Nayi Subah, Ek Nayi Asha, Ek Nayi Umeed, Ek Nayi Soch, Ek Baar Phir Asman Chhune ki Koshish, Kamayab Hone ki Chahat - Naye Saal ki Subah ki Pehle Kiran ke Saath……

Happy and Prosperous New Year

